# Tea and other diet-related practices in relation to sleep health in midlife women from Mexico City: qualitative and quantitative findings

**DOI:** 10.3389/frsle.2024.1477046

**Published:** 2024-11-27

**Authors:** Astrid N. Zamora, Elizabeth F. S. Roberts, Lilian Sharp, Catherine Borra, Jennifer Lee, Martha M. Téllez-Rojo, Karen E. Peterson, Libni A. Torres-Olascoaga, Alejandra Cantoral, Erica C. Jansen

**Affiliations:** ^1^Department of Nutritional Sciences, University of Michigan School of Public Health, Ann Arbor, MI, United States; ^2^Department of Epidemiology and Population Health, Stanford University School of Medicine, Stanford University, Stanford, CA, United States; ^3^Department of Anthropology, University of Michigan, Ann Arbor, MI, United States; ^4^Social Research Institute, University College London, London, United Kingdom; ^5^Center for Research on Nutrition and Health, National Institute of Public Health, Cuernavaca, Mexico; ^6^Health Department, Ibero-American University, Mexico City, Mexico; ^7^Department of Neurology, Division of Sleep Medicine, Michigan Medicine, Ann Arbor, MI, United States

**Keywords:** diet, epidemiology, ethnography, midlife women, nutrition, qualitative research, sleep duration, tea

## Abstract

**Purpose:**

Little is known regarding women's lived experiences of how diet impacts sleep. Based on ethnographic interviews among working-class women from Mexico City, our primary aim was to identify themes related to diet and sleep among midlife women. Informed by qualitative analyses, a secondary aim was to examine associations between tea and sleep duration in a broader cohort.

**Materials and methods:**

We conducted a cross-sectional study that entailed in-depth ethnographic interviews about sleep and other behaviors, including diet, with a purposive sample of 30 women from the ELEMENT cohort. Ethnographer field notes and transcripts were analyzed using thematic analysis. Guided by findings from the interviews demonstrating that tea consumption might be associated with sleep, we conducted *post-hoc* analyses of the relationship between tea and sleep duration using data from food frequency questionnaires and actigraphy, respectively, in the broader cohort (*n* = 406).

**Results:**

The mean (SD) age of the ethnographic sample was 50.0 (9.0) years. The top noted theme was the use of herbal tea (in Spanish *infusion*) to improve sleep; most women (29/30) discussed herbal teas, characterizing them as a “natural remedy” to facilitate sleep. The mean (SD) age of the broader sample (*N* = 406) was 48.4 (6.2) years. *Post-hoc* analyses revealed positive associations between tea without sugar (though not necessarily herbal tea) and sleep duration. We found that every serving of tea without sugar consumed was associated with an 18.0 min per night [β (SE) = 18.0 (7.8); *p* = 0.022] and a 13.4 min per night [β (SE) =13.4 (5.6); *p* = 0.017] increase in weekend and 7-day sleep duration, respectively.

**Conclusions:**

Within a sample of 30 midlife women, dietary practices were described in relation to sleep, specifically the consumption of herbal teas to promote sleep.

## 1 Introduction

Recent epidemiologic evidence has revealed positive associations between healthy dietary patterns and improved sleep quality (Flor-Alemany et al., 2020; Zuraikat et al., 2020; Zhu et al., 2021), and these associations may be even stronger among women than men (Jansen et al., 2021). For example, data from pre/peri-menopausal women residing in the United States (US) who participated in the National Health and Nutrition Examination Study (NHANES) revealed that lower intake of protein, carbohydrates, and other important nutrients was significantly associated with a higher risk for very short sleep (Zhu et al., 2021). Other longitudinal epidemiological studies have found that diets rich in fruits and vegetables and those more highly aligned with the Mediterranean diet were associated with a lower likelihood of insomnia and poor sleep quality among midlife women (Castro-Diehl et al., 2018; Jansen et al., 2020).

While there is an existing body of research on the connection between healthy diets and sleep, we know very little about the dietary strategies midlife women use to enhance their sleep. Existing studies have largely concentrated on have primarily focused on adolescents (Kira et al., 2014; Quante et al., 2018; Vandendriessche et al., 2022) and patient populations (Stremler et al., 2011). For example, a study conducted in the US explored adolescents' food-related sleep strategies, such as consuming milk before bedtime or avoiding late-night meals (Quante et al., 2018). In contrast, a study of Australian adults noted a general lack of participant awareness or inquiry into the relationship between lifestyle factors like diet and sleep (Liang and Ploderer, 2016). Moreover, very few studies focus on Latin American populations, which are likely to differ in social and environmental context in regards to diet and sleep than previously studied populations.

Prior studies by our team have demonstrated associations between diet and sleep, including a positive correlation between a fruit and vegetable-based diet and improved sleep quality among midlife women from Mexico (Jansen et al., 2020). However, the prior study did not examine how women used or avoided certain foods or beverages to aid in sleep or other connections between diet and sleep present in their everyday lives. This highlights the need for qualitative research, including ethnography, which involves long-term participant observation and in-depth interviews to discern what matters within a particular social ecology (Goodson and Vassar, 2011). Employing ethnographic methods can provide deeper insights into the lived experiences of study participants, ultimately guiding the more effective design of research studies and questions.

Enhancing our understanding of midlife women's experiences with diet and sleep has the potential to impact public health by informing interventions that target women's health, dietary practices, and sleep quality, particularly during the midlife transition. First, findings may provide knowledge related to how dietary patterns and diet-related practices affect sleep health, which could inform dietary recommendations that target sleep. Additionally, insights from this work may lead to novel exploration of dietary behaviors that have not been fully considered in previous epidemiological research studies. Understanding lived experiences is important when designing public health recommendations that are not only scientifically appropriate, but also culturally and practically relevant for midlife women. Ultimately, results from this work may provide knowledge required to develop interventions that improve both diet and sleep for midlife women, especially those residing in regions with limited access to sleep health resources. The primary aim of this study was to understand experiences of how diet influences sleep among midlife women from Mexico City, using ethnographic data from interviews about sleep.

## 2 Materials and methods

### 2.1 Sample and recruitment

Participants were initially enrolled in the Early Life Exposure in Mexico to Environmental Toxicants (ELEMENT) birth cohort starting in 1994 when they were pregnant or after delivery. Women were primarily recruited from the Mexican Institute of Social Security family clinics, which cater to a low- to middle-income population in Mexico City, MX. A subsequent midlife follow-up study (*n* = 587) was conducted between 2019 and 2023 (with a pause from March 2020 through late 2021 due to COVID-19). During this visit, women provided comprehensive data on demographic characteristics, anthropometry, and general health. Sleep was measured via 7-day actigraphy and sleep diaries. Women who participated in the in-person visit prior to the pandemic were eligible to engage in ethnographic interviews. From this pool of eligible women, we used a purposive sampling strategy to recruit 30 women, seeking a diverse sample of women across the age spectrum from 30 to 64 years—a factor hypothesized to influence sleep and menopause (the primary focus of the study). Recruitment was carried out over the phone in Spanish by a member of the ELEMENT fieldwork team—who called women in the eligibility pool and provided comprehensive details about the study to gauge interest. Women who indicated they were interested were then scheduled for an interview. The ELEMENT fieldwork team continued making phone calls until 30 women were enrolled and consented. Thus, the final ethnographic sample comprised 30 interviewees from the quantitative sample. Recruitment and interviews took place between June and September 2021, focusing on gathering nuanced data about women's experiences during pre-, peri-, and post-menopause. A participation rate of 100% was achieved for the participants who were approached to participate in the qualitative study.

The Research, Ethics, and Biosafety Committees of the Mexico National Institute of Public Health (INSP) and the Human Subjects Committee at the University of Michigan approved all research protocols and procedures. All participants provided informed consent. Specifically, for ethnographic interviews, participants were required to provide verbal informed consent at the commencement of each interview.

### 2.2 Qualitative interviews and ethnographic data collection

For this cross-sectional study, two ELEMENT researchers conducted ethnographic phone interviews in Spanish, including ANZ, who is fluent in Spanish and received training in ethnographic fieldwork methods from an anthropologist (EFSR). The ethnographic interviews, lasting ~45–60 min, were facilitated through Zoom audioconferencing (San Jose, CA, USA), and the audio recordings were securely stored for subsequent transcription.

The interview protocol encompassed a range of pivotal topics concerning sleep health throughout different life stages, with a specific focus on current sleep health. There were three particular questions from the interview protocol that prompted participants to articulate their dietary habits in connection with their sleep or knowledge of how diet may impact their sleep or the sleep of others. Given existing research that has demonstrated that dietary factors (e.g., food, herbs. etc.) can have an impact on sleep without even being consumed, for example, via inhalation of lavender (Lillehei et al., 2015) and smelling food (Gaeta and Wilson, 2022), participants in our study could describe experiences with food without direct consumption. Some questions included additional probes (only used if necessary to enrich the interview) related to sleep and were formulated as follows:

1) What types of things affect your sleep and the sleep of other people in general? *Probes:* Does eating or drinking something before bed affect your sleep? Light or noise? Other environmental factors? Toxic chemicals? Smoking cigarettes or consuming alcohol? Pain, stress, anxieties, or other physical factors?
2) What advice have you heard or know to help someone fall asleep?
3) Have you ever taken medications or remedies to help you sleep? If so, which ones?

To ensure anonymity in the written record, a native Spanish speaker transcribed the interviews verbatim, and a research team member systematically replaced all names and identifiable information with pseudonyms.

### 2.3 Analysis

To characterize the ethnographic sample, we include a summary of demographic characteristics (i.e., age, household socioeconomic status). Depending on the covariate, we present either median and interquartile range (IQR) or proportion (%).

The initial phase of our analysis involved a thorough review of interview transcripts, which was undertaken by multiple members of our interdisciplinary research team (LS, CB, and ANZ), specifically searching for excerpts where participants discussed sleep remedies—conceptualized here as any intervention perceived to facilitate sleep. Once these excerpts were identified, illustrative excerpts were extracted from the transcripts and logged into Microsoft Excel for a more detailed and concentrated interpretative analysis. Using thematic analysis (Boyatzis, 1998), we then identified thematic clusters and associated sub-themes inductively. Next, the primary analyst (ANZ) conducted a line-by-line coding of the extracted qualitative data. These codes served to categorize participants' experiences of sleep remedies and, importantly, captured multiple mentions of sleep remedies within one extracted excerpt. To ensure the fidelity of our interpretations, we undertook a process of triangulation, with two researchers (ANZ and ECJ) collectively evaluating and discussing the evolving coding framework, which was guided by thematic analysis. This process of articulating and cross-examining the coding process continued until a consensus was reached for all themes and sub-themes. In the presentation of our findings, we provide the overarching themes and sub-themes, the frequency of their occurrence, and verbatim quotes (translated from Spanish to English by ANZ) to illuminate the richness and complexity of experiences and practices related to food and beverage-related sleep remedies.

### 2.4 *Post-hoc* epidemiological analysis

After completing qualitative analysis for the primary aim, results revealed that tea consumption emerged as a prominent theme in our qualitative analysis. Based on this finding, we carried out *post-hoc* analyses of the FFQs to examine the link between usual tea consumption and objectively assessed sleep duration within the broader cohort of midlife women (*N* = 406 women with both sleep and dietary measurements), of which the sample of women that completed ethnographic interviews (*N* = 30) was included. Below, we describe data collection efforts and statistical analysis associated with the *post-hoc* epidemiologic analysis.

#### 2.4.1 Data collection

##### 2.4.1.1 Food frequency questionnaires

Tea consumption was assessed based on two items from a semi-quantitative food frequency questionnaire from Mexico's National Institute of Public Health National Health and Nutrition Survey (Hernández-Avila et al., 1998), which was adapted from the Willett Food Frequency Questionnaire (FFQ; Willett et al., 1985). The two items included: “tea or infusion (herbal tea) with sugar” and “tea or infusion (herbal tea) without sugar.” Trained social workers administered the FFQ, employing visual aids and measuring utensils (e.g., spoons and cups) to assist participants in identifying foods and portion sizes. The estimated daily intake of tea with and without sugar was calculated, with servings measured at 240 mL per day. In addition, using the FFQ, we computed total energy intake using the residual method, as described in detail elsewhere (Willett et al., 1997).

##### 2.4.1.2 Actigraphy-based sleep duration

Sleep duration was estimated using wrist-actigraphy devices (ActiGraph GT3X+; ActiGraph LLC, Pensacola, FL) that participants wore on the non-dominant wrist for seven consecutive days following the study visit. Trained personnel placed an actigraphy device on the participant's wrist at the end of the study visit. Weekend nightly sleep duration (a measure of the mean total sleep duration at night from Friday through Sunday) was estimated from actigraphy data using a pruned dynamic programming (PDP) algorithm developed by R (R Foundation for Statistical Computing, Vienna, Austria). The PDP approach incorporates self-reported bedtimes and wake times to improve accuracy (Baek, 2021). Three sleep duration measures were examined: weekday (Monday through Thursday), weekend (Friday through Sunday), and total sleep duration (7-day average).

#### 2.4.2 Covariates

For the *post-hoc* analyses covariates included age, household socioeconomic status, smoking status, alcohol consumption behavior, moderate-vigorous physical activity, and menopause status. Household socioeconomic status (SES) was self-reported and assessed using a 10-item region-specific household-based survey that was developed and index standardized (i.e., AMAI 8 × 7) by the Mexican Association of Marketing Research and Public Opinion Agencies (AMAI) (López Romo, 2009) to classify the SES of the Mexican population. Classification of smoking status and alcohol consumption behavior have been previously described (Zamora et al., 2021). Moderate-to-vigorous physical activity (MVPA; min/week) was determined via wrist actigraphy using the Hildebrand cutoffs (Hildebrand et al., 2014) within the R package GGIR.

#### 2.4.3 Analysis

We ran linear regression models with sleep duration (weekday, weekend, and total as separate models) as the outcome and tea consumption (no sugar and with sugar as separate models) as the continuous exposure. Model 1 (base model) was unadjusted and provided crude estimates for these associations; model 2 adjusted for age, socioeconomic status, total energy intake, moderate-vigorous physical activity (across weekdays and weekends), alcohol consumption, and smoking status. Statistical significance was determined a priori at *p* < 0.05. All analyses were conducted using SAS 9.4 (Cary, NC, USA).

## 3 Results

Women in the ethnographic sample were, on average, 50 years of age (SD = 9; Table 1). Moreover, the mean (SD) age for the broader cohort sample was 48.4 (6.2) years. From a visual inspection of the table, we observed differences across characteristics between the ethnographic study sample and the broader cohort sample. For instance, household SES, where the ethnographic sample was split between lower vs. middle/higher SES, while the broader sample primarily fell into the middle/higher SES group (50 vs. 61.5%). Similarly, the ethnographic sample had a nightly longer sleep duration compared to the broader sample for total sleep duration (7 days) and weekday sleep duration, while the weekend sleep duration was the same across both samples (mean = 7.1 h per night).

**Table 1:** Description of the ethnographic and broader cohort samples of midlife women from the ELEMENT study.

	**Ethnographic study sample**	**Broader cohort study sample**
	**(*****N*** = **30)**	**(*****N*** = **406)**
	**Mean (SD) or %**	
Age	50.0 (9.0)	48.4 (6.2)
Household socioeconomic status (SES)		
*Lower*	50.0	37.7
*Middle/higher*	50.0	61.5
*Missing*	0.0	0.8
Total sleep duration, hours per day^a^	7.2 (1.3)	6.6 (1.2)
Weekend sleep duration, hours per day^a^	7.1 (1.5)	7.1 (1.6)
Weekday sleep duration, hours per day^a^	7.6 (1.9)	6.8 (1.1)

^a^Data from actigraphy device.

Based on interview transcripts, the overarching themes that emerged regarding diet and sleep could be divided into food- vs. beverage-related practices (Table 2). Regarding food-related practices, women often talked about either not eating before bed or only eating light meals. One specific food that women talked about eating before bed (which could be classified as a “light meal”) was lettuce. Women also talked about placing lettuce or bay leaves under the pillow at night to help them fall asleep.

**Table 2:** Ethnographic interview food and beverage-related practices that impact sleep among a sample of midlife women from the Mexico City-based ELEMENT cohort (*N* = 30).

**Diet and sleep themes**	***N* (number of women who mentioned the topic)**	**Representative quotes from transcripts**
**Food-related**		
Don't eat before bed	4	“No, I try not to eat like that when I'm going to sleep, I try to have my last meal at eight at night” (Int 1)
Light meal/snack/dinner	6	“If you eat something that is light so that you don't lose weight, it won't make you sleepy” (Int 26)
Eat lettuce	2	“Having a glass of tea, milk at night or eating lettuce” (Int 23)
Bay leaves under pillow/lettuce under pillow	2	“Well, I have heard to put a few leaves of lettuce under your pillow and you will see that you will sleep well” (Int 25)
Lettuce bath	2	“No, it's like when babies can't sleep, they say to give them a bath with lettuce and then they will relax...” (Int 2)
**Beverage-related**		
Tea	29	“What I have done sometimes is drink seven blossoms tea and that is the one that allows me to sleep” (Int 4)
Milk	9	“A glass of warm milk, but well I would advise more with the glass of water” (Int 17)
Coffee	10	“Many say not to drink coffee because you won't be able to sleep, but coffee never kept me awake, really, no, no, not that, I don't know why, but coffee doesn't keep me awake” (Int 12) “I love having my cup of coffee with milk… but coffee keeps me from being able to sleep, it scares away my feelings of being sleepy” (Int 18)
Whiskey with milk	1	“ Well, these are home remedies… they're grandma's remedies as some like to say… I've heard about drinking whiskey with milk…” (Int 2)

Total mentions, participant could have mentioned multiple times in one interview.

ELEMENT, Early Life Exposures in Mexico to ENvironmental Toxicants; Int, interview; N, frequency mentioned.

Beverage-related practices were the most discussed diet and sleep topics. Notably, most participants (29/30) discussed herbal teas, characterizing them as a “natural remedy” to facilitate sleep. In addition to herbal teas, milk was frequently cited as a sleep-promoting beverage, with a singular mention of an unconventional concoction of whiskey with milk as a nocturnal aid. Conversely, there were contrasting views on coffee. While coffee consumption was recurrently mentioned as an integral element of some women's night-time routine, its impact on sleep was predominantly categorized as neutral (i.e., does not affect sleep). However, a subset of participants highlighted its potential to disrupt sleep, demonstrating varied individual responses to caffeine consumption.

A closer look into the interview transcripts revealed that 16 different categories of tea were mentioned, with most tea types being herbal, except for green tea (Table 3). The most described tea among the sample was orange blossom tea (“té de azahares”), while other notable herbal teas mentioned included chamomile, passionflower, other fruit teas, and lettuce tea.

**Table 3:** Ethnographic interview mentions of teas that promote sleep.

**Types of teas mentioned**	N^*^
Tea (té)	7
Green tea (té verde)	1
Chamomile tea (té de manzanilla)	1
Wormwood tea (té de ajenjo)	2
Orange blossom tea (té de azahares)	5
Cinamon tea with warm milk (té de canela con leche tibia)	1
Seven Flowers (té de siete flores)	1
Lettuce tea (té de lechuga)	1
Tomato tea (té de tomate)	1
Lemon tea (té de limon)	2
Relaxing or linden tea (té relajante o de tila)	1
Orange tea (té de naranjo)	1
Lemon balm tea (té de toronjil)	1
Valerian tea (é de valeriana)	1
Peppermint tea (té de hierba buena)	1
Passionflower tea (té de pasiflora)	2
Total mentions of tea	29

^*^N, frequency mentioned.

The frequency of tea in the interview transcripts (qualitative data), led us to conduct *post-hoc* epidemiological analysis of tea consumption and sleep duration (quantitative data) with the broader dataset of women. *Post-hoc* results from unadjusted and fully adjusted regression models revealed several statistically significant associations between consumption of tea without sugar with weekend and total (7-day) sleep duration before and after adjusting for potential confounders (Table 4). For instance, after full adjustment, results revealed that every additional 240 mL serving of tea without sugar consumed per day was associated with an 18.0 min per night [β (SE) = 18.0 (7.8); *p* = 0.022] increase in weekend sleep duration. The magnitude was highly similar for total sleep duration [β (SE) = 13.4 (5.6); *p* = 0.017]. However, no statistically significant associations were revealed between tea with sugar or total tea consumed with any of the three sleep duration measures.

**Table 4:** Associations between tea consumption with weekday, weekend, and total sleep duration among sample of a midlife women from the Mexico-City-based ELEMENT cohort (*n* = 406).

	**Weekday sleep duration, min/night**		**Weekend sleep duration, min/night**		**Total sleep duration, min/night**	
	β **(SE)**	***P***	β **(SE)**	***P***	β **(SE)**	***P***
**Tea without sugar per serving (*****N*** = **406)**						
Model 1	12.0 (6.1)	0.050	17.3 (7.8)	0.026	13.6 (5.6)	0.015
Model 2	11.4 (6.2)	0.067	18.0 (7.8)	0.022	13.4 (5.6)	0.017
**Tea with sugar per serving (*****N*** = **406)**						
Model 1	−4.8 (8.3)	0.562	−11.9 (10.6)	0.260	−7.5 (7.6)	0.321
Model 2	−4.3 (8.3)	0.603	−10.5 (10.6)	0.325	−7.0 (7.5)	0.354
**Total tea**^a^ **per serving (*****N*** = **406)**						
Model 1	7.2 (5.4)	0.180	8.3 (6.8)	0.223	7.3 (4.9)	0.137
Model 2	6.7 (5.4)	0.211	9.4 (6.9)	0.173	7.1 (4.9)	0.145

ELEMENT, Early Life Exposures in Mexico to ENvironmental Toxicants; SE, standard error; Model 1, unadjusted; Model 2, adjusted for age, socioeconomic status, and energy intake to account for overall dietary patterns, physical activity indicators, separately accounting for moderate and vigorous activities across weekdays and weekends, alcohol consumption, and smoking status.

^a^Total tea = tea without sugar + tea with sugar.

## 4 Discussion

Our study had two key findings: (1) that midlife women in Mexico City drink herbal tea to facilitate sleep and (2) that drinking herbal tea is associated with longer sleep duration among a broader cohort of this study population. More specifically, this study identified key practices surrounding diet and sleep that Mexican women in their midlife years know and/or practice. There were two primary focuses on their dietary strategies relating to sleep: avoidance of eating before bed or opting for light meals and particular attention to beverage consumption, especially milk, coffee, and, most commonly, herbal tea. Given the prominent role of tea consumption, a subsequent *post-hoc* epidemiological analysis was undertaken to examine this association in more detail. Results from this analysis revealed a positive connection between consumption of tea without sugar and sleep duration, illustrating that each additional serving of tea without sugar was associated with ~14-min increased nightly sleep duration.

Previous empirical research among Mexican women has demonstrated that diet patterns are associated with sleep. Within a different cohort of Mexican women, dietary patterns rich in fruits and vegetables and lower in fast food and red meat were associated with prospectively assessed better sleep quality (Jansen et al., 2020). Furthermore, individual beverages and food from the dietary patterns, including alcohol, coffee, and soda, were related to worse sleep, while milk/yogurt intake was linked to better sleep. Within the present study, some of the same items emerged in ethnographic interviews related to diet and sleep, including alcohol, coffee, soda, and milk. Additionally, about one-third of the ethnographic sample described dietary practices before bed, including not eating before bed or eating a light meal only. This practice has also been documented in previous literature; for example, experimental studies have shown that nocturnal eating (i.e., food intake 30–60 min before bedtime) is negatively associated with sleep quality, with a greater effect in generally healthy women than in generally healthy men (Crispim et al., 2011). Similarly, consuming light meals or snacks rather than larger meals in the hours leading up to bedtime could be beneficial for sleep since they are easier to digest (Benton et al., 2022). Interestingly, one of the unexpected foods that several participants mentioned was lettuce, either consumed as a light bedtime provision or used as a non-consumable sleep aid. Lettuce as a sleep aid shows up in pop culture (e.g., TikTok videos) and the gray literature (The Truth About the Viral Lettuce Water TikTok Hack, 2021; Let Us Talk About Lettuce Water, 2023). However, it has not been thoroughly investigated in epidemiological studies except for a few experimental studies that showed the sleep-inducing effect of lettuce varieties and the extracts in animals (Kim et al., 2017; Ahn et al., 2023) and one human study of people with insomnia showed improvements in sleep rating scales among patients who received lettuce seed oil (Yakoot et al., 2011).

Within our ethnographic interviews on diet and sleep, the predominant theme that emerged was the role of beverages on sleep. We found that the women used different teas (primarily herbal) was embedded to enhance sleep-−29 of the 30 women in our sample talked about tea in some way. For some women, consuming tea was described as part of their sleep routine, which may indicate an appreciation for the calming act of tea preparation (Chen, 2002) rather than the tea itself. Others described the sleep-enhancing effects of specific teas (Kim et al., 2018), especially orange blossom tea. In line with these descriptions, some existing studies among mostly aging adults have provided evidence of positive associations between frequent tea drinking and improved sleep quality (Unno et al., 2017; Ouyang et al., 2022; Wei et al., 2023). However, to our best knowledge, no existing studies have specifically explored the impact of herbal tea consumption on sleep quality among midlife women, other than one existing study on tea and sleep quality in post-menopausal women, which found a positive association (Mahmoudi et al., 2020). Our findings about the prevalence of herbal tea consumption to enhance sleep led us to investigate whether the consumption of tea, as reported in food frequency questionnaires from a broader cohort, was associated with sleep duration. Intriguingly, findings from our *post-hoc* epidemiological analysis mirrored the qualitative findings, revealing a positive association between consuming tea without sugar and nightly sleep duration. These congruent findings suggest the need for further research into the relationships between herbal tea and sleep among midlife Mexican women and may indicate a potential role for herbal tea within interventions to improve sleep in this population. Moreover, this finding highlights the value of using qualitative data to guide hypothesis generation and quantitative data analysis within cohort studies.

Findings from our *post-hoc* analyses generally mirror existing literature on herbal tea consumption and sleep. However, most studies have focused on sleep quality and not duration. For example, a double-blind, placebo-controlled study investigated the effects of *Passiflora incarnata* (passionflower) herbal tea on subjective sleep quality and found that consumption of a low dose of *Passiflora incarnata*, in the form of tea, yields short-term subjective sleep benefits (Ngan and Conduit, 2011). A separate study by Baek et al. found that herbal tea treatment improved subjective sleep quality in a sample of 20 adults (men and women; Baek et al., 2018). However, to our knowledge, no studies have specifically examined the relationship between herbal tea consumption and sleep duration using actigraphy. This gap in the literature highlights the need for further research utilizing objective sleep measures. Moreover, while the exact mechanism linking herbal tea consumption and longer sleep duration remains unclear, several possibilities may explain the association. For instance, herbal teas are known to contain amino acids and polyphenols that can reduce inflammation and provide antioxidant effects, which could enhance both sleep quality and duration (Hibi, 2023; Xiang et al., 2023). Additionally, herbal tea may aid in metabolic regulation by stabilizing blood glucose levels (Schiano et al., 2021). Finally, herbal tea could replace the consumption of other beverages known to be harmful to sleep, such as sugar-sweetened beverages, which are linked to sleep difficulties (Jansen et al., 2022). Further research is needed to fully understand the underlying mechanisms.

Although not as frequently mentioned as tea, another beverage-related practice that emerged from a few interviews was the consumption of milk as a sleep aid. Some experimental studies have provided evidence of the sleep-inducing properties of tryptophan, an amino acid present in milk (Qian et al., 2021; Ortega et al., 2023). A recently published systematic review showed an inconsistent association between milk consumption and sleep quality among human populations but found evidence that dairy consumption overall may be beneficial for sleep (Komada et al., 2020). Moreover, within a Mexican adolescent cohort, higher milk consumption was associated with longer sleep duration, but this was specific to boys (Jansen et al., 2022). Future work that investigates the timing of milk consumption relative to bedtime and its preparation in relation to sleep is needed.

Women shared mixed and nuanced views concerning the role of coffee on sleep. Some participants did not associate coffee with disrupted sleep and instead described drinking it as part of their night-time routine. Another subset of women identified coffee as a potential sleep barrier, which is in line with the majority of research on this topic (Claydon et al., 2023; Gardiner et al., 2023). These conflicting views could reflect personal differences in the metabolism of caffeine, as research indicates that sensitivity to caffeine is variable across populations (Nehlig, 2018; Barcelos et al., 2020). Interestingly, there was almost no mention of the role of soda or alcohol on sleep. This is possibly related to the fact that these beverages are not commonly consumed before bed in this population of women—a hypothesis that could be tested in future work within the broader cohort.

### 4.1 Strengths and limitations

Our study had many strengths. A notable strength of this study is the focus on an underserved demographic—midlife Mexican women—and their diet-sleep experiences. A second significant strength was the open-ended nature of qualitative inquiry, which provided a vehicle for participants to articulate their realities, experiences, and interpretations. This qualitative richness, integrated with the *post-hoc* quantitative sleep data, allowed for a new finding that herbal tea consumption may be associated with longer sleep. The insights gleaned from the ethnographic data laid the groundwork for designing future diet and sleep-related studies to investigate sleep remedies previously underappreciated in the epidemiological literature. This primarily includes tea consumption but also suggests the need for further investigation into milk, lettuce, coffee, and the meals consumed before bed. We must also acknowledge the limitations of this study. First, while the ethnographic sample was a subset of the broader cohort, we observed minor differences between the two. These included differences in mean sleep duration from actigraphy and household socioeconomic status (SES). The broader sample had more participants from middle to higher SES, while the ethnographic sample was more evenly split between lower and middle/higher SES. These distinctions, though minor, suggest that the ethnographic sample may not fully represent the broader study cohort. Another limitation arose due to the COVID-19 pandemic, which impeded our ability to conduct in-person ethnographic fieldwork (originally designed to be collected within households), thus limiting our insights into how participants navigate sleep relative to their habitual behaviors, daily routines, and household environments. In interpreting our *post-hoc* findings related to tea consumption, it is important to acknowledge the limitations of the FFQ utilized in our study. The FFQ did not provide detailed information on the specific types of teas consumed, including the distinction between herbal infusions or black tea or the caffeine content of the teas. This lack of specificity in the FFQ data should be considered when interpreting the results regarding tea consumption patterns. As with any cross-sectional research design, our study is limited in its ability to establish causal relationships between variables. Moreover, regression models did not adjust for the intake of sleep medications, which could have confounded the relationship between tea consumption and sleep duration in the broader cohort. Cross-sectional studies provide a snapshot of data at a single point in time, making it challenging to conclude the direction of relationships or causality. Future longitudinal studies or intervention trials would be valuable in elucidating the temporal and potentially causal relationships observed in our cross-sectional analysis. Finally, because we focused on midlife women from Mexico City, caution must still be exercised in generalizing these findings to other demographic groups. It should also be noted that while the *post-hoc* analysis included data from the broader cohort study of midlife women from Mexican City, the findings from the ethnographic interviews are reflective of a specific group of women and may not necessarily represent all Mexican women.

## 5 Conclusion

In our ethnographic interviews, we explored the relationship between diet (e.g., food, beverages, etc.) and sleep. While our questions were intended to be very open-ended, the participants frequently brought up tea consumption. The fact that tea became a prominent theme without a specific prompt regarding tea was illustrative in itself. This highlights the importance of gathering data on diet and sleep behaviors through open-ended questions, allowing themes to emerge without a preset survey. In the case of this study, we found that the majority of women mentioned herbal tea as a sleep-enhancing strategy. It is important to note that tea consumption, while commonly discussed, was not the primary focus of our inquiry. Rather, it emerged as a reflection of participants' concerns regarding diet and sleep. This emphasizes the complex and subjective nature of the diet-sleep relationship, which warrants further exploration in future research to disentangle the effects of specific dietary components. Future work should investigate the role of specific teas and practices related to tea consumption, as well as milk, lettuce, and night-time meals or snacks, in relation to sleep health among midlife women.

## Data Availability

The datasets present in this article are available upon reasonable request to the senior author, Erica C. Jansen, janerica@umich.edu.
